# Efficacy of BioMin F and NovaMin toothpastes against streptococcus mutans: an in vitro study

**DOI:** 10.1038/s41405-024-00202-x

**Published:** 2024-03-11

**Authors:** Haya Doura Alomari, Yasser Alsayed Tolibah, Chaza Kouchaji

**Affiliations:** Department of Pediatric Dentistry, Faculty of Dentistry, Damascus University, Damascus, Syrian Arab Republic

**Keywords:** Dentistry, Oral diseases

## Abstract

**Objective:**

This in vitro study was accomplished to demonstrate the antibacterial efficacy of BioMin F and NovaMin toothpastes against the recently-isolated Streptococcus Mutans in comparison with a commonly used fluoride toothpaste.

**Materials and methods:**

Dental plaque collection method was adopted to isolate streptococcus mutans in children with dental caries. Then an ideal Streptococcus Mutans colony was incubated in 20 Petri dishes that contained Mueller-Hinton medium. Each dish had 3 wells; one well for each toothpaste (BioMin F, NovMin, and Signal) to perform the agar diffusion test. After incubating for 24 hours, the inhabitation zone around each well of each Petri dish was noticed and measured. Statistical Analysis was achieved using a statistical package, SPSS Windows version 17, by applying Kruskal-Wallis with Mann–Whitney U test (α = 0.05).

**Results:**

BioMin F showed the highest mean of inhibition zone diameter ($\bar{x}$ = 2.67 mm) in compared with NovaMin and Signal ($\bar{x}$ = 0.39 mm and $\bar{x}$ = 2.19 mm; *p* < 0.001 in each pairwise comparison).

**Conclusion:**

BioMin F toothpaste showed superior antibacterial effect against Streptococcus mutans to Signal and NovaMin toothpastes. Novamin showed the lowest antibacterial effect. This in vitro study suggests that BioMin F toothpaste shows encouraging potential to be recommended as a preventive measure to reduce the caries risk.

## Introduction

In Syria, as in third-world countries, dental health has reached a low level with a high prevalence of dental caries especially in children. Therefore, the focus was on the mechanism of dental caries and ways to prevent it to reduce the spread of this disease [1].

The mechanism of dental caries is somewhat complex, as it results from the accumulation of plaque on the teeth surface, which in turn takes place in biochemical reactions that ferment carbohydrates, which leads to the production of acid that destroys the enamel and then spreads into dentinal tubes [2]. One of the essential opportunistic pathogens of dental caries is Streptococcus mutans, which has acidogenic properties and the ability to fabricate an extracellular matrix [3].

Several Studies have revealed that daily oral hygiene practices, like toothbrushing, are impressive in controlling the dental caries process [4]. Nevertheless, for individuals at high risk of dental caries, the combination of antibacterial and remineralizing agents with oral care products is mandatory [5, 6].

Bioactive glass (BAG) toothpaste is considered one of the materials used in oral health support because of its role in remineralizing the demineralized enamel and fluoride content [7]. The BAG is mainly consisting of 45% SiO4, 24.5% CaO, 24.5% Na2O, and 6% P2O5, thus it produces Hydroxyl Carbonate-Apatite, which is crystalline in nature and is comparable to the tooth construction [8]. Moreover, BAGs have significant advantages like they can raise the pH of the oral medium due to calcium and phosphorus ions released [9], and they can generate apatite, potentially addressing any marginal gaps resulting from polymerization shrinkage after composite restorations [10].

BAG has experienced a lot of improvements and changes under different commercial names like NovaBone, NovaMin, NovaThera, and BioMin [11, 12].

BioMin can penetrate the dentin canals and deposit on the surface of the tooth by its special small-sized BAG particles. BioMin F, which contains fluorine in its composition, was introduced in 1990. It can form fluorapatite when it is diffused in saliva and can continue to release fluorine up to 12 h after brushing [13].

NovaMin consists of calcium sodium phospho-silicate which is the active ingredient that enables it to bind to the surface of the tooth to initiate the process of enamel remineralization when its components are in contact with saliva or any aqueous media [14]. NovaMin is reactive when exposed to body fluids, where it quickly releases calcium, sodium, and phosphate ions. There is a localized transient increase in pH due to the release of sodium, which helps the calcium and phosphate which exist in the NovaMin toothpaste to form a calcium phosphate layer [15].

Various studies have been accomplished to test the efficacy of BioMin and NovaMin toothpaste in enamel remineralization [16] and their clinical effects to reduce the sensitivity after teeth bleaching [17], but, to the best of our knowledge, no attempt has been made to test their antimicrobial efficacy against Streptococcus mutans. Therefore, the aim of future research would be to assess the bacteriostatic effect of various pastes containing bioactive glasses. Thus, this in vitro study aimed to compare the effect of BioMin F, and NovaMin toothpastes against Streptococcus mutans in comparison with a commonly used fluoride toothpaste like Signal. The null hypothesis assumed that all toothpastes would limit the development of Streptococcus mutans in the same way in vitro.

## Material and methods

### Ethical statement

This experimental in vitro study was conducted respecting the ethical guidelines of the Declaration of Helsinki. The research project was ethically approved by the Local Research Ethics Committee of the Faculty of Dentistry, Damascus University (UDDS-693-07092020/SRC-1450), and was funded by Damascus University (funder No. 501100020595).

### Preparing the culture medium of mitis salivarius agar

The Petri dishes that contained the selective medium were previously prepared during the samples collecting period, by dissolving 90 g/L of Mitis Salivarius Agar (M-S Agar) (HiMedia, Maharashtra, India) in distilled water and adding 20% sucrose, then the mixture was placed in a wet sterilizer (JSAT-85; JSR®, Chungchungnam-Do, South Korea) at a temperature of 110 °C and a pressure of 15 psi for 15 min. When the mixture was cooled to 50 °C, the suspension of 0.2 IU/ml bacitracin (Fagron, Glinde, Germany) was added, then the medium was poured into sterile Petri dishes and they were left to cool. Afterward, they were put in the refrigerator upside down to prevent water condensation on the surface of the medium. The previous method of preparing the M-S Agar was similar to the method described by Tanzer [18].

### Streptococcus mutans isolation with dental plaque collected technique

Ten children diagnosed with Severe Early Childhood Caries (SECC), aged between 2 and 6 years, were invited to participate in dental plaque collection. The children’s parents were given all the information about the aim of this procedure and signed the informed consent. The study population was 4 (40%) males and 6 (60%) females.

Study swaps were collected away from meals and teeth brushing for at least two hours. Plaque was collected from both the lingual surfaces of the lower primary second molars and the buccal surfaces of the upper primary second molars using moist heat-sterilized toothpicks in a wet sterilizer (JSAT-85; JSR®, Chungchungnam-Do, South Korea) for 24 hours. Afterward, the samples were transferred into Eppendorf tubes (Seal-Rite® Scientific, Inc., Ocala, FL, USA) that contained 1 ml of sterile normal saline. Samples were homogenized for 1 min to detach the material collected from the swab. Then, a dilution of each sample was made by adding 0.1 ml of the tube containing the plaque to another sterile tube containing 0.9 ml of sterilized normal saline, then the tube was shaken for homogenization. Then 0.1 ml of the previous solution was added to another tube containing 0.9 ml of sterilized normal saline, which represents the second dilution, and it was repeated until the fifth dilution.

The samples were cultured within an hour of the sample collection period on Petri dishes containing Mitis Salivarius Bacitracin Agar from the final suspension of each sample, 50 μL was planted on a Petri dish numbered with the sample number. To ensure equal distribution over the entire surface of the agar, the brushing process was repeated in all directions while rotating the dish. After 15 min the dishes were turned over to ensure that the medium absorbed the bacteria, and then the dishes were incubated upside down at 37 °C for 72 h in a 15% CO2 incubator (Bacteriological Incubator 6640-01-071- 6596/National Appliance Heinicke Co. Tualatin, USA). The previous method of Streptococcus mutans isolation was comparable to the method described by Motisuki [19]. The ideal Streptococcus mutans colonies appear as small, rough, and heaped on the agar surface, and may appear as a bright bubble due to excess glucan synthesis by sucrose [20]. Both catalase tests (which was negative) and Gram stain (which was positive) were performed on model colonies of Streptococcus mutans produced after incubation. Moreover, it was confirmed that the isolated samples were Streptococcus mutans by conducting polymerase chain reaction tests (LightCycler® PRO System; Roche, Basel, Switzerland) within the Atomic Energy Authority, Damascus, Syria, which were positive for Streptococcus mutans. Then, a copy of the isolated samples was kept in the laboratories of the College of Science, Department of Botany, within the heart-brain infusion medium at −80 °C [21], to be used later in the stage of testing the sensitivity of Streptococcus mutans to the materials studied in this research.

### Bacterial activation and preparing the study dishes

Before 48 h of toothpaste application, the Streptococcus mutans were activated by culturing on Petri dishes containing Mueller-Hinton medium, then a model colony was taken after 24 h incubation, and incubated again in a liquid nutrient medium tube (Brain Heart Infusion (BM0070; EO Labs, Bonnybridge, Scotland)) and placed in the incubator at 37 °C for 24 h to have the active Streptococcus mutans sample. The samples were removed from the incubator, and the tube was shaken and stirred using the vortex device (CSL-VORTEX, Thistle Scientific Ltd, Glasgow, United Kingdom) for one minute to obtain complete mixing of the components within the tube.

Streptococcus mutans density was determined at 0.5 of McFarland standard (1.5×10^8 CFU/mL) using PhonexSpec (BD PhoenixSpec™ nephelometer 440910/ Becton, Dickinson and Co., Maryland 21152 USA), where a quantity of medium containing Streptococcus mutans was taken from the previous tube and gradually added to a tube containing sterile physiological serum until getting the required concentration.

The agar was cooked from Mueller-Hinton medium and poured into 20 petri plates (60 mm diameter), then left to harden at room temperature.

For the test, three wells with a diameter of 9 mm were made in each plate over the entire thickness of the agar with a depth of approximately 4 mm through a sterile puncher. The controlled turbidity bacterial solution was shaken using the vortex device for 30 s, 100 μl of this suspension was then taken using a micropipette to be scattered on the plate and handed out over the entire surface of the agar through the tip of a sterile glass tube with a Z-shaped movement left and right, then the dish was rotated 60 degrees with repeating the same movement, then it was rotating another 60 degrees with the same movement. Finally, the bacterial suspension was spread by brushes with circular movement on the edges of the dish to ensure that it spread uniformly over the entire plate, then the dishes were left for 20–30 minutes at room temperature to allow the absorption of the bacterial suspension. The previous methods of both Streptococcus mutans activation and study dish preparation were comparable to those described by Prasanth [22].

### Evaluation of toothpastes antibacterial effect with agar diffusion method

The toothpastes used in the study are presented in Table 1.

**Table 1:** The toothpastes used in the present study.

Toothpates	Composition according to manufacturer	Manufacturer
BioMin F	Glycerin, Silica, Poley Ethelene Glycol 400, FluoroCalciumPhosphoSilicate, Sodium Lauryl Sulfate, Titanium Dioxide, Aroma, Carbomer, and Potassium Acesulfame. Fluoride concentration is 530 µg/g by weight	BioMin Technologies Ltd, Stoke-on-Trent, Uk
NovaMin	NovaMin 5.0% w/w Calcium Sodium Phosphosilicate (Bioactiveُ Glass 45s5), Sodium Fluoride (1450 ppm) (0.2299% w/w) (fluoride 0.104% w/w), Fluoride, Glycerin, Poley Ethelene Glycol 8, Silica Hydrated, Titaniumُ Dioxide, Carbomer, Cocamidopropyl Betaine, Sodium Methyl Cocoyl Taurate, D-Limonene, Aroma, Silica, Sodium Saccharin	GSK Sensodyne; Brentford, Middlesex, United Kingdom
Signal	Sodium Monofluorophosphate (1450 ppm Fluoride), Calcium Carbonate, Aqua, Sorbitol, Sodium Lauryl Sulfate, Silica, Aroma, Potassium Citrate, Cellulose Gum, Trisodium Phosphate, Phenylcarbinol, Limonene, Sodium Saccharin.	Unilever, Ho Chi Minh City, Vietnam

**Fig. 1:**
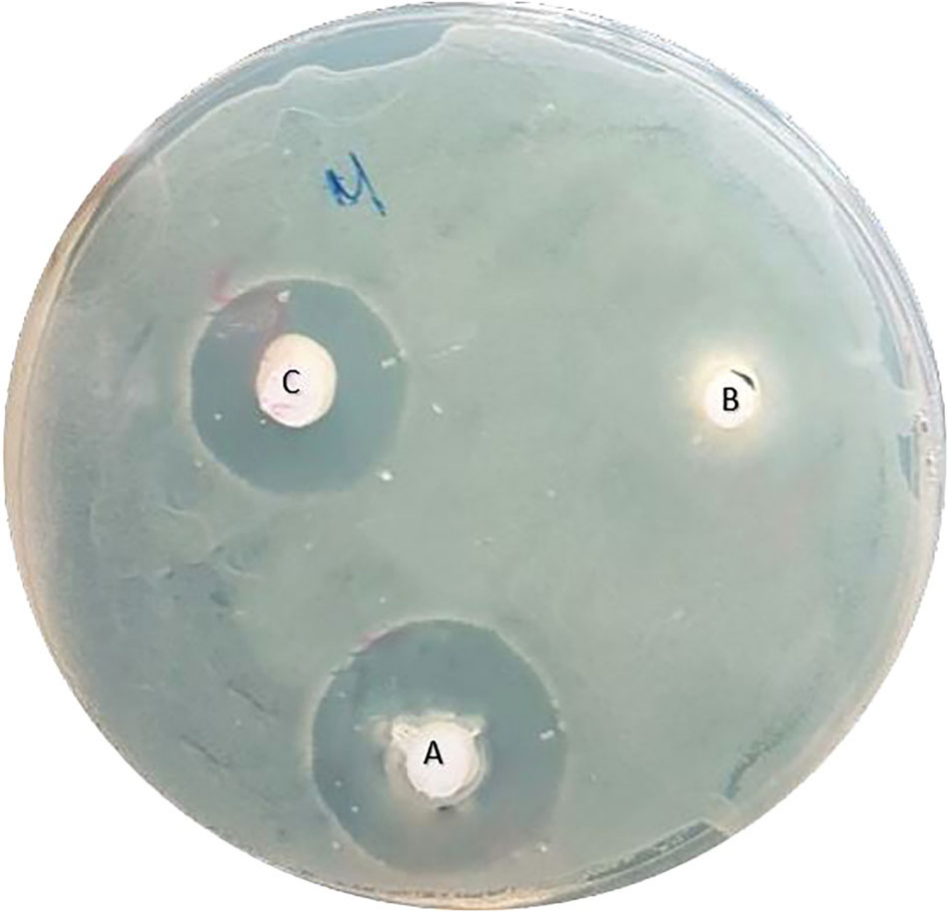
A petri dish after 24 h of incubating; (**A**) BioMin F well, (**B**) NovaMin well, and (**C**) signal well.

The tested toothpaste; BioMin F, (BioMin Technologies Ltd, Stoke-on-Trent, UK), NovaMin (GSK Sensodyne; Brentford, Middlesex, United Kingdom), and Signal (Unilever, Ho Chi Minh City, Vietnam) were filled with sterile insulin syringes before being used immediately to work more easily and applied within the specified wells in the dish. Each toothpaste was placed in each well and the wells were coded on the back of the dish, and each dish was coded with a specific number, then dishes were incubated upside down at 37 ^o^C for 24 h in a 15% CO_2_ incubator. Inhibition zones surrounding each toothpaste were observed by clearance of bacterial colonies around the well (Fig. 1). Thereafter, the circle was measured in millimeters with a digital caliper (WEN Digital Caliper, Performance Tool - Wilmar LLC, Kent, Washington, USA), a higher inhibition zone diameter meant a higher antibacterial activity of the tested mixture.

The previous methods of evaluating the anti-bacterial capacity of the toothpaste with the agar diffusion method were comparable to those described by Prasanth [22].

### Statistical analysis

Descriptive statistics and statistical analysis were accomplished with SPSS 17 (Statistical Package for Social Science, SPSS, version 17.0, SPSS, Chicago, IL, USA). Shapiro–Wilk test showed a normal distribution of inhibition zone diameters of the toothpaste’s wells. The One-way ANOVA test was used for intergroup comparisons, and the Bonferroni test was used for pairwise comparisons. A *p* value < 0.05 was considered significant and had a confidence level of 95%.

## Results

The Inhibition zones of each of the three toothpastes (BioMin F, NonaMin, and Signal) wells was examined separately in 20 petri dishes after 24 h of incubation.

The descriptive statistics of the range mean, standard deviation, and the One-way ANOVA test result was shown in Table 2, where there were significant differences between the three groups.

**Table 2:** Comparison of inhibition zones diameters between groups.

Group	*N*	Range	Mean ± standard deviation	^*P* value
BioMin F	20 wells	2.0–3.9 mm	2.67 mm ± 0.42	<0.001*
NovaMin	20 wells	0.0–1.0 mm	0.39 mm ± 0.42	
Signal	20 wells	2.0–3.1 mm	2.19 mm ± 0.26	

^:One-way ANOVA test.,

*Significant difference.

The Bonferroni test was used to detect differences in pairwise comparisons, which showed that, BioMin F group had the highest inhibition zone diameters ($\bar{x}$ = 2.67) with significant different in comparison with NovaMin and Signal groups ($\bar{x}$ = 0.39 and $\bar{x}$ = 2.19 respectively; *p* < 0.001 in each pairwise comparisons). Moreover, Signal group showed higher inhibition zone diameters with significant different in comparison with NovaMin group (*p* < 0.001).

## Discussion

Dental caries is a prevalent chronic non-communicable disease associated with multiple microorganisms especially Streptococcus mutans [23]. Similar to most chronic diseases, if interceptive measures are taken to control the causative factors, such as daily mechanical and chemical toothbrushes, the initiation and advancement of dental caries can be better controlled especially in high-risk populations [2, 6].

With the advent of new toothpaste such as bioactive glass toothpaste, which was characterized by their ability to remineralize carious lesions in permanent and primary teeth [24, 25].

Due to the absence of stored pre-isolated Streptococcus mutans colonies, this study was bifurcated into two parts. The first one delineates the isolation of Streptococcus mutans by researchers from dental plaque collected from 10 children with SECC, aiming to identify a representative Streptococcus mutans colony for subsequent culture in the petri dishes employed in the present study. The second one aimed to assess the ability of both BioMin F and NovaMin toothpaste to inhibit the bacterial action of recently isolated Streptococcus Mutans, compared to a commonly used fluoride toothpaste like Signal.

The specific culture medium Mitis Salivarius Bacitracin was used to in vitro isolate Streptococcus mutans, where it is characterized by giving accurate results because it allows obtaining a good quantity of Streptococcus mutans when they are present in low numbers within the sample [26].

The agar diffusion test can be used when the quality of the antibacterial agents cannot be evaluated with chemical methods, as it depends on the direct proportional relationship between the inhibition zone of the tested microorganisms and the concentration (dose) of the studied agent placed on the agar microbial medium [27, 28]. However, it is appropriate only for diffusive materials to determine their antibacterial activity, and the main limitation of this test is the inability to locate whether the material is bactericidal or bacteriostatic, and may affect its reliability in determining some dental toothpaste antibacterial characteristics [29].

According to the results of the current study, the null hypothesis was rejected, as they suggest that BioMin F toothpaste had a significantly higher ability to inhibit the Streptococcus mutans action compared to NovaMin and Signal toothpaste. Moreover, Signal toothpaste showed a significantly higher ability to inhibit the Streptococcus mutans action compared to NovaMin, where the last one had a very weak inhibition ability.

The reason may be due to the presence of fluoride ions in the bio-glass structure beside calcium and phosphate ions (fluor-hydroxyapatite) in BioMin F, which allows longer gradually delivery of Fluoride especially in the neutral environment [30]. Fluoride is widely used as an anti-cariogenic bacterial factor such as Streptococcus mutans [31]. Moreover, it affects the bacterial cell membrane and acts as a metabolic inhibitor while protons acidify the cytoplasm, resulting in the death of the bacteria [32].

Additionally, as it was found in an in vitro study, the crystalline structure consisting of calcium and phosphate has an inhibitory effect on the formation of Streptococcus mutans biofilm [33]. Moreover, according to the BioMin manufacturer, the fluor-hydroxyapatite that is formated by BioMin F is ten times more resistant to acid dissolution in comparison with the hydroxyapatite formulated with NovaMin [34].

Both NovaMin and Signal toothpaste, which were used in the current study, had Sodium Fluoride (1450 ppm) which loses its therapeutic effect during 90–120 minutes, while the fluorhydroxyapatite that formolate by BioMin F can release the Fluoride up to 12 hours [34]. Additionally, the study by Dang and colleagues assumes that the short-term effect of Fluoride does not affect the activity of Streptococcus mutans [35]. The previous factors may explain the superiority of BioMin F paste over NovaMin and Signal toothpaste in inhibiting Streptococcus mutans.

The superiority of the Signal toothpaste over the NonaMin toothpaste may be explained by the existence of Sodium Lauryl Sulfate in the Signal paste, which has been proven in an in vitro study to have anti-Streptococcus mutans properties after two days of incubation in Petri dishes [36].

Dai and colleagues assessed the anti-cariogenic bacteria effect of a new bioactive glass powder named Huaxi bioactive glass-ceramic (HX-BGC) which contains calcium, phosphate, fluoride, and strontium ions, and after scanning electron microscopy and confocal laser scanning microscopy detection, they revealed that HX-BGC had inhibited the acid production and growth of Streptococcus mutans, Streptococcus sobrinus, Lactobacillus acidophilius, and Lactobacillus rhamnosus [37]. Although this study’s mythology is different, our result met this study, the reason may be due to the affinity of the components of BioMin F paste with the components of the powder used in the study.

On the other hand, Stoor and his colleagues evaluated the ability of the S53P4 bioglass powder against some oral bacteria, including Streptococcus mutans, in the bacterial suspension. Although the previous bioglass didn’t contain fluoride, they found that it had good antibacterial properties against Streptococcus mutans after incubating the bacteria for 60 min and counting the bacterial colonies. The main difference between the results of the previous study and the current study may be due to the use of a different study methodology, in addition to the different incubation period, as the previous bioglass may become resistant to Streptococcus mutans within only a short period [38].

This study had two limitations, the first one was assessing the anti-bacterial effects of BioMin F, NovaMin, and Signal toothpastes against Streptococcus mutans only, where there was great difficulty and cost to isolate it, and it was possible to study the anti-bacterial effects of previous toothpastes against other cariogenic bacteria such as Streptococcus sobrinus, Lactobacillus acidophilus, and Lactobacillus rhamnosus. The second one was the study of the antibacterial effect in Mueller-Hinton medium only, as this medium is characterized by its moderate acidity (7.2–7.4) [39], and it was possible to study the anti-bacterial capacity of the previous toothpaste in a more acidic medium, as the fluoride in the acidic medium is more active, and can better interfere with acid production and proliferation of bacteria [31]. Another limitation stems from the lack of alignment between the bacteria sourced from each child’s mouth and the Petri dishes utilized to gauge the antibacterial effects of the three toothpastes. This highlights the imperative for subsequent studies to rigorously evaluate the antibacterial efficacy of these toothpastes against the diverse bacterial strains found within the plaque of children afflicted with SECC separately to find the best anti-bacterial toothpaste.

## Conclusion

After 24 h of incubation in the Mueller-Hinton medium, BioMin F toothpaste showed superior antibacterial effect against Streptococcus mutans to Signal and NovaMin toothpastes. Novamin showed the lowest antibacterial effect. This in vitro study suggests that BioMin F toothpaste shows encouraging potential to be recommended as a preventive measure to reduce the caries risk.

## Data Availability

The datasets used and/or analysed during the current study available from the corresponding author on reasonable request.
